# Selection signatures on the autosomes and the X chromosome in the prolific Belclare sheep breed

**DOI:** 10.1016/j.vas.2026.100659

**Published:** 2026-04-15

**Authors:** Julia Lisboa Rodrigues, Flavio Schramm Schenkel, Larissa Graciano Braga, Ana Carolina Almeida Rollo de Paz, Rafael Nakamura Watanabe, Noirin McHugh, Donagh Pearse Berry, Marcos Eli Buzanskas, Danísio Prado Munari

**Affiliations:** a Centre for Genetic Improvement of Livestock, Department of Animal Biosciences, University of Guelph, Canada; b Departamento de Ciências Exatas, Universidade Estadual Paulista (UNESP), Faculdade de Ciências Agrárias e Veterinárias, Jaboticabal, Brazil; c Animal & Grassland Research and Innovation Center, Teagasc, Moorepark, Fermoy, Co, Cork, Ireland; d Departamento de Melhoramento e Nutrição Animal, Universidade Estadual Paulista (UNESP), Faculdade de Medicina Veterinária e Zootecnia, Botucatu, Brazil

**Keywords:** Haplotype analysis, Male fertility, *Ovis aries*, Precocity, Reproductive traits, Selective sweep

## Abstract

Sheep (*Ovis aries*) is a poly-ovulatory species, with litter size varying considerably within and among breeds. Litter size reflects ewe prolificacy, and prolific breeds represent valuable genetic resources for the sheep industry. Belclare is a composite sheep breed developed primarily for reproductive performance and used for meat production. Artificial selection for beneficial alleles related to reproductive traits can create detectable genomic patterns known as selection signatures (SS). This study aimed to detect selection signatures on autosomes and the X chromosome in Belclare sheep and annotate candidate regions associated with reproductive traits. A total of 8421 ewes genotyped with the OvineSNP50 BeadChip were analyzed. Breeds were classified into two groups: prolific (Belclare) and less prolific (Beltex, Charollais, Suffolk, and Texel). Inter-population selection signatures were detected using Cross-Population Extended Haplotype Homozygosity (XP-EHH) and Fixation Index (FST) in 100-kb non-overlapping windows across autosomes (top 1%) and the X chromosome (top 10%). Overlapping signals formed regions ranging from 200–400 kb. Using XP-EHH, regions of 400 kb were detected on OAR3 and OAR5, while one shared SS between XP-EHH and F_ST_ was identified on OARX. The genes within these regions were associated with reproductive traits in both ewes and rams. X-linked genes, including *TAF7L, ATP1B4*, and *ZMYND12*, were associated with sperm motility. Genes involved in ovarian processes, including follicular development, ovulation, and early pregnancy, such as *MID1IP1, IL1RAPL2*, and *EDN2*, were also identified within the SS regions. Overall, the SS detected in Belclare elucidated the genetic basis underlying its prolificacy relative to less prolific breeds.

## 1 Introduction

Sheep (*Ovis aries*) is a poly-ovulatory species, with litter sizes varying considerably, ranging from 1 to 9, within and among breeds (Pokharel et al., 2018). Litter size reflects an ewe's prolificacy, with prolific breeds showing higher ovulation rates and larger litters than less prolific breeds (Abdoli et al., 2016). Prolific breeds are valuable genetic resources for the sheep industry, with larger litters contributing to greater farm profitability (Pokharel et al., 2018). Several genes have been identified as associated with prolificacy, facilitating stronger selection pressure in particular flocks (Abdoli et al., 2016). Belclare is a composite sheep breed developed in Ireland under intense selection for high prolificacy, with meat production as a secondary objective. The breed emerged from the sheep genetics programme established by the Agricultural Institute (An Foras Talúntais) in the 1960s (Hanrahan, 2004).

Belclare was developed by crossing a High Fertility line established in the late 1960s (e.g., ewes that had given birth to four or more lambs or produced nine or more lambs over three consecutive seasons) with several other breeds. Based on pedigree records, its genetic composition includes Texel (37.5%), Lleyn (25%), Finnish Landrace selected for high ovulation rate (19.5%), Galway (9%), and small contributions from Cheviot (2.5%), Suffolk (1.5%), and other local Irish breeds (4%) (Hanrahan et al., 2004). As a result, Belclare sheep have higher ovulation rates and litter sizes than many traditional meat breeds, and mutations in the *BMP15, GDF9*, and *BMPR1B* genes have been reported to be associated with these reproductive traits through DNA sequencing analyses (Hanrahan et al., 2004; Liu et al., 2014).

Artificial selection for regions containing beneficial alleles, such as those related to reproductive traits, can increase their frequencies and fix them within the population, leaving detectable patterns in the genome known as selection signatures (Saravanan et al., 2020). The genomic consequences of this selection pressure can differ between autosomes and sex chromosomes. According to Rubin et al. (2012), the X chromosome should be analyzed solely for the detection of selection signatures, preferably utilizing only females, due to the differing effective population sizes and selective forces affecting sex chromosomes and autosomes across genders. The effective population size of the X chromosome is three-quarters of the autosomes and undergoes more genetic drift than the autosomes (Heyer & Segurel, 2010). Furthermore, limiting the analysis to females avoids the confounding effects of male hemizygosity on the X chromosome. The X chromosome in sheep harbors several genes associated with desirable breeding traits, suggesting it is a good target for detecting selection signatures (Zhu et al., 2015).

High-throughput sequencing and single-nucleotide polymorphism (SNP) genotyping technologies, advanced bioinformatics tools, and robust statistical tests have all created new opportunities to explore and understand these genomic selection patterns (Saravanan et al., 2020). Methods for identifying selection signatures between populations can be divided into single-site differentiation, such as the fixation index (F_ST_) (Wright, 1949), and haplotype-based differentiation, such as cross-population extended haplotype homozygosity (XP-EHH) (Sabeti et al., 2007). Using multiple methods can improve the likelihood of detecting selection signatures (Qanbari and Simianer, 2014). Nonetheless, strategies based on different statistical assumptions may detect complementary or overlapping signals, and results across methods can complement each other to provide supporting evidence for candidate regions. Selection signatures in Belclare have been previously investigated by Purfield et al. (2017) and Rodrigues et al. (2025) using methods such as iHS, Tajima’s D, and Runs of Homozygosity (R_OH_). However, these studies did not include the X chromosome and did not compare this highly prolific breed with a combined group of less prolific sheep breeds.

Therefore, this study aimed to identify selection signatures on the autosomes and the X chromosome in Belclare sheep by comparing this prolific breed with less prolific breeds, and to perform gene annotation and QTL enrichment of candidate regions associated with reproductive traits.

## 2 Material and methods

### 2.1 Samples, genotyping, and quality control

Approval from the Animal Care and Use Committee was not required, as the genotypes were obtained from the Irish national sheep breeding program database hosted by Sheep Ireland (http://www.sheep.ie). Single nucleotide polymorphism (SNP) genotype data from the Illumina OvineSNP50 panel were available for five sheep breeds and consisted of 2444 Belclare, 146 Beltex, 1500 Charollais, 1241 Suffolk, and 3399 Texel. Phenotypes and pedigree databases were not available for this study. The studied breeds were raised in Ireland and are included in the Irish breeding program that focuses on meat production traits (Bohan et al., 2019). Among them, only Belclare is considered a prolific meat sheep breed.

Quality control was performed across all breeds using PLINK v1.9 (Purcell et al., 2007) to detect selection signatures. Samples and SNPs with genotyping call rates below 95% were excluded, along with SNPs with minor allele frequency (MAF) < 0.01. The SNPs located on autosomal and X chromosomes were both considered in the present study, and their positions were defined according to the sheep (*Ovis aries*) genome assembly ARS-UI_Ramb_v2.0 (Davenport et al., 2022). Haplotype phasing was performed for each breed independently using BEAGLE v.4.1 (Browning et al., 2018) using the default parameters and the imputation feature disabled (*impute=false*). After quality control, a total of 47,887 SNPs were retained for analyses, comprising 46,643 autosomal SNPs and 1244 X-linked SNPs, from 8421 ewes.

### 2.2 Principal component analysis

Principal component analysis (PCA) was performed using PLINK v1.9 (Purcell et al., 2007) to assess genetic relationships among breeds. The same quality control described above was applied to the PCA. Eigenvectors computed for each animal were used to construct scatter plots along the first two principal components (i.e., PC 1 and PC 2), which explained 37.12% and 19.23% of the total variance for the first and second principal components, respectively.

### 2.3 Linkage disequilibrium decay

Linkage disequilibrium (r^2^) among SNPs was estimated within each sheep breed using PLINK v1.9 (Purcell et al., 2007). The r^2^ quantifies the extent of LD between pairs of loci (Hill, 1981). The estimation was performed within non-overlapping 1-Mb windows, considering all r^2^ values between marker pairs (*–ld-window-r2 0*).

### 2.4 Detection of selection signatures

Prior to selection signature detection, the ewes were classified into two groups: (i) a prolific group, which includes Belclare, and (ii) a less prolific group, including Beltex, Charollais, Suffolk, and Texel, which were pooled into a single analytical group. The classification of prolific and less prolific breeds was based on litter size and their historical use in breeding programs. Belclare, well known as a prolific breed in Ireland, has a litter size of approximately 2.2 under typical on-farm management (Keady & Hanrahan, 2022), and has been historically used to increase prolificacy through crosses with less prolific breeds such as Texel and Suffolk. Two approaches were used to detect the selection signatures, including the Cross-Population Extended Haplotype Homozygosity (XP-EHH) (Sabeti et al., 2007) and the Fixation Index (F_ST_) (Wright, 1949), to increase the power of selection signatures detection.

### 2.5 Cross-population extended haplotype homozygosity (XP-EHH)

The XP-EHH is based on the extended haplotype homozygosity (EHH). Cross-population EHH compares the integrated EHH across 2 populations at a given SNP and identifies selected alleles that have approached, or achieved, fixation in one population but not the other (Braga et al., 2025). This method is indicated for detecting ongoing or nearly fixed selection signatures because it is based on LD and identifies alleles that have rapidly increased in frequency and remain associated with nearby polymorphisms (Sabeti et al., 2007). The XP-EHH values were calculated with the REHH v.3.2.2 R package (Gautier & Vitalis, 2012; Gautier et al., 2017) in non-overlapping 100 kb windows, with a minimum of two SNPs per window. This method produces directional scores: positive values indicate selection in the first population, and negative values indicate selection in the second.

### 2.6 Fixation index (F_ST_)

The fixation index (Wright, 1949) is a test that measures population genetic differentiation and can detect actual genetic variants under selection. The F_ST_ values range from zero, indicating no difference in allelic frequencies between the two populations, to one, meaning that each population is fixed for a different allele (Weir and Cockerham, 1984). The F_ST_ was calculated using the *–fst* function using PLINK v1.9 (Purcell et al., 2007). The F_ST_ values were then summarized in non-overlapping 100 kb windows, with a minimum of two SNPs per window, using the *calc_candidate_regions* function implemented in the R package REHH v.3.2.2 (Gautier & Vitalis, 2012; Gautier et al., 2017). Higher F_ST_ values indicate divergent selection pressures acting on a given locus between the populations (Saravanan et al., 2020).

### 2.7 Selection of candidate selection signatures

Regions under positive selection in Belclare were defined as the top 1% of windows on autosomes and the top 10% on the X chromosome, based on the highest values from each method (XP-EHH and F_ST_), using Belclare ewes as the reference population. The different thresholds reflect differences in evolutionary dynamics: the X chromosome is subject to stronger genetic drift than autosomes, with lower SNP density, resulting in smaller absolute test statistic values. Applying a stringent threshold (top 1%) for the X chromosome would greatly reduce the power to detect candidate regions. Selection signature regions were identified separately for autosomes and the X chromosome, and separate lists were compiled for each method, which were then used for gene annotation and functional enrichment analysis.

### 2.8 Gene annotation and functional enrichment analysis

The gene and quantitative trait loci (QTL) annotation was performed within the selection signatures using the R package GALLO v.1.5 (Fonseca et al., 2020). The ovine reference genome ARS-UI_Ramb_v2.0 (Davenport et al., 2022) and the SheepQTL database (https://www.animalgenome.org/cgi-bin/QTLdb/OA/index) (Hu et al., 2022) were used in the gene and QTL searches, respectively. Gene enrichment analysis was performed using the gprofiler2 R package (Kolberg et al., 2020) with a False Discovery Rate (FDR) threshold of < 0.10. Gene Ontology (GO) and metabolic pathways were retrieved from the Kyoto Encyclopedia of Genes and Genomes (KEGG) database.

## 3 Results

### 3.1 Principal component analysis

**Fig. 1:**
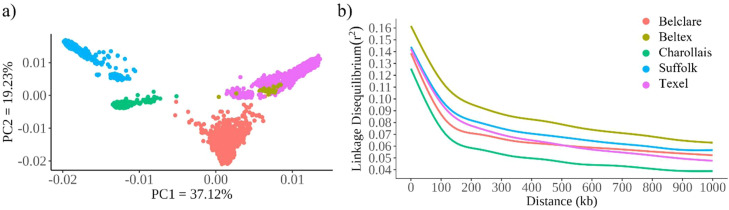
Population genetic analyses of sheep breeds. (a) Principal component analysis of five sheep breeds. (b) Decay of linkage disequilibrium within five sheep breeds.

The first two principal components of the PCA explained 56.35% of the total variance in the genetic relationships among the five sheep breeds (Fig. 1a). Beltex overlapped with Texel, whereas Charollais, Suffolk, and Belclare formed three distinct clusters. Suffolk showed two subclusters within the same breed. Belclare did not overlap with Texel. However, it was the closest to the Texel cluster.

### 3.2 Linkage disequilibrium decay

The mean r^2^ for all sheep breeds within a range of 100 kb to 1-Mb window is shown in Supplementary Table S1. The average r^2^ at 100 kb for Belclare, Beltex, Charollais, Suffolk, and Texel was 0.109, 0.136, 0.096, 0.118, and 0.116, respectively (Supplementary Table S1). Beltex showed the slowest decay in LD, followed by Suffolk and Texel breeds (Fig. 1b), and they had the highest number of SNP pairs with r^2^>0.20 (Supplementary Table S1).

### 3.3 Selection signature detection

**Fig. 2:**
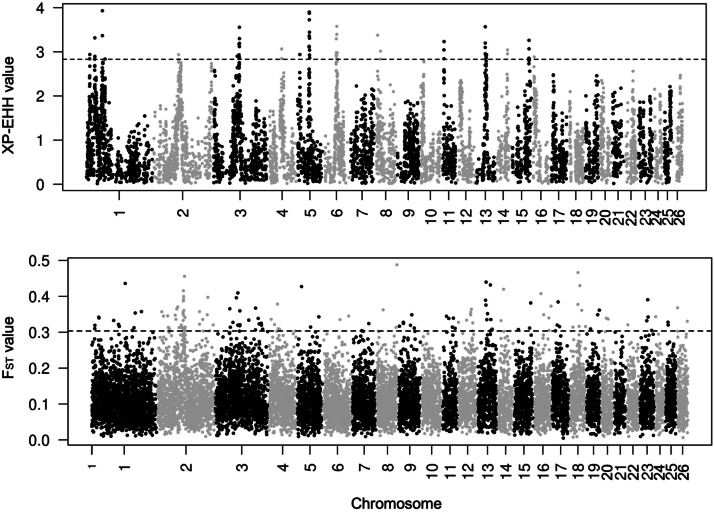
Manhattan plots of XP-EHH and F_ST_ values across autosomes, calculated in 100-kb non-overlapping windows, comparing Belclare with a group of less prolific meat sheep breeds. The horizontal line indicates the threshold corresponding to the top 1% of windows.

**Fig. 3:**
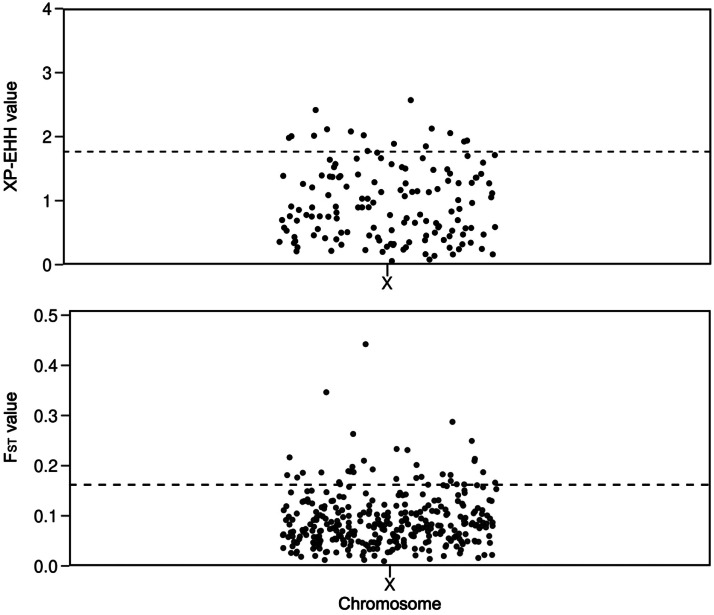
Manhattan plots of XP-EHH and F_ST_ values on the X chromosome, calculated in 100-kb non-overlapping windows, comparing Belclare with a group of less prolific meat sheep breeds. The horizontal line indicates the threshold corresponding to the top 10% of windows.

Selection signature regions in the Belclare breed were detected across the genome, and the number of regions, genes, and QTL within the positive selection in the autosomes and X chromosome is presented in Table 1. Among all chromosomes, OAR2 (Ovis aries chromosome 2), OAR3, OAR1, OAR13, OAR14, and OAR5 exhibited the highest number of selection signatures detected by XP-EHH and F_ST_. Selection signatures covered 0.82% of the autosomes (21.6 Mb) and 4.13% of the X chromosome (5.9 Mb). The percentages were calculated based on the total length of the autosomal (2.65 Gb) and X chromosome (142.9 Mb) assemblies used in this study. The distribution of XP-EHH and F_ST_ values across the autosomes and X chromosome is presented in Fig. 2, Fig. 3, respectively. The candidate genes and QTL located within selection regions in Belclare are listed in Supplementary Tables S2 and S3, respectively. Selection signature regions overlapped and formed longer segments, ranging from 200–400 kb. Using the XP-EHH method, lengths of 400 kb were formed in OAR3 and OAR5 (Supplementary Table S2). One common selection signature region between XP-EHH and F_ST_ was detected on OARX (109.2–109.3 Mb), within which the protein gene TENM1 and the ENSOARG00020023883 miRNA are located.

**Table 1:** Number of selection signature regions detected using the XP-EHH and F_ST_ methods, and the genes and QTLs annotated within these regions.

Method	Autosomes			X Chromosome		
	SS	Genes	QTL	SS	Genes	QTL
XP-EHH	40	88	26	12	18	2
F_ST_	137	268	58	33	48	13
Total*	177	356	84	44	66	15

SS: Number of top selection signature regions detected on autosomes and the X chromosome after merging adjacent windows; XP-EHH: Cross-Population Extended Haplotype Homozygosity; F_ST_: Fixation index;.

⁎ Total: number of unique regions after merging adjacent windows and removing overlapping regions between methods.

Common genes identified within the selection signature regions between methods are shown in Table 2. The results of functional enrichment analysis of genes and QTLs within the selection signature regions are shown in Supplementary Tables S4 and S5, respectively. Annotated genes were enriched in functional categories related to neuro-signaling and synaptic transmission, oxidative stress and redox homeostasis, intracellular signaling and epigenetic regulation, membrane transport processes, and X-linked genetic architecture. Among the enriched QTLs, three were associated with reproductive traits, including two related to offspring number (OAR11 and OARX) and one associated with reproductive seasonality (OARX).

**Table 2:** Common genes detected within selection signature regions by XP-EHH and F_ST_ methods.

Chromosome	Method			Gene ID	Gene name
	F_ST_ (Mb)		XP-EHH (Mb)		
X		98:0–98.2	98.1–98:2	ENSOARG00020019925	
X		107.2–107.3	107.3–107.4	ENSOARG00020022286	*GRIA3*
X		109.2–109.3	109.2–109.3	ENSOARG00020023417	*TENM1*
X		109.2–109.3	109.2–109.3	ENSOARG00020023883	
15		76.4–76.5	76.5–76.6	ENSOARG00020028322	

Mb: Megabase; XP-EHH: Cross-Population Extended Haplotype Homozygosity; F_ST_: Fixation index. Genes identified only by ENSOARG accession numbers correspond to loci not yet assigned official gene symbols in the reference annotation database.

## 4 Discussion

### 4.1 Principal component analysis and linkage disequilibrium decay

Population structure within and between breeds can be explored using PCA, and LD decay can provide insights into selection and historical breeding patterns. In this study, the PCA revealed genetic similarities between Texel and Beltex breeds, consistent with findings reported by O’Brien et al. (2020). This relationship is expected, as the Beltex breed was developed in Belgium through selective breeding of Texel sheep for muscular hypertrophy (O’Brien et al., 2020). Consistently, LD decay analysis showed that Beltex presented higher and more persistent r² values across genomic distances. According to Kijas et al. (2014), breeds with lower genetic diversity exhibit higher mean LD and slower LD decay, which in this case may reflect the intensive artificial selection applied to achieve the desired hypertrophy in Beltex. However, demographic factors may also contribute to this pattern. Charollais and Suffolk formed distinct clusters in the PCA. The two sub-clusters observed within Suffolk may be explained by the historical importation of New Zealand Suffolk animals into Ireland (Featherstone et al., 2021), as also reported by O’Brien et al. (2020) and Rodrigues et al. (2025).

Belclare formed a distinct cluster from the less prolific breeds included in this study. Although Texel contributed to the development of Belclare, the breed showed genetic differentiation from Texel and the other analyzed breeds. This pattern may be associated with the intensive selection for prolificacy in Belclare. The LD decay pattern in Belclare showed the lowest r² values and the most rapid decline among the breeds, consistent with Rodrigues et al. (2025). In addition to LD decay, these authors also reported greater genetic diversity in Belclare, which may explain the faster LD decay observed in this breed compared with breeds primarily selected for meat production. These results highlight the genetic differentiation between Belclare and the less prolific breeds, supporting the comparative approach used in this study to investigate selection signatures associated with prolificacy.

### 4.2 Selection signatures detection in the prolific meat breed

The detection of selection signatures between different populations can provide a better understanding of how and why those populations differ. In this study, comparing a prolific breed with less prolific breeds aims to identify genomic regions potentially under positive selection for prolificacy in Belclare. None of the previously reported mutations in *BMP15, GDF9*, and *BMPR1B* genes, such as FecX^G^, FecX^B^, FecG^H^, and FecG^F^, which have been associated with high ovulation rate in Belclare sheep (Liu et al., 2014), were detected within the selection signatures in the present study. The absence of FecX^G^, FecX^B^, and FecG^H^ within the selection signature regions may be because ewes homozygous for those mutations exhibit abnormal ovarian development and are sterile (Notter, 2008), and such phenotypes were not observed in the studied animals. On the other hand, Belclare ewes homozygous for FecG^F^ are not sterile and exhibit a higher ovulation rate than heterozygotes, with frequency reported to range from 0.17 to 0.18 based on DNA sequence data (Mullen & Hanrahan, 2014).

X-linked genes identified within selection signature regions were associated with reproductive traits in sheep. The *GRIA3* gene, detected using both F_ST_ and XP-EHH, has previously been reported to be associated with total productive life, somatic cell score, and daughter pregnancy rate in Holstein cattle and has been proposed as a candidate gene for longevity (Cole et al., 2011; Zhang et al., 2021). Ewe longevity is related to reproductive efficiency, reflecting how long ewes remain productive in the herd. This trait is economically important, as increasing ewe longevity reduces culling rates and replacement costs in breeding flocks (McLaren et al., 2020). Therefore, selection for reproductive performance in Belclare may be associated with genes related to longer productive life, potentially providing additional economic benefits to sheep breeders worldwide.

Several genes identified on the X chromosome have previously been associated with reproductive traits in both males and females. Genes such as *KDM5C, ATP1B4, SMARCA1, TAF7L*, and *RPL36A* have been linked to male reproductive traits, particularly sperm motility and fertility, whereas *MID1IP1, KIF4A, FOXO4, NLGN3, GABRQ, GABRA3*, and *IL1RAPL2* have been associated with female reproductive processes.

The *ATP1B4* gene, identified using XP-EHH, encodes a subunit of ion pumps that play essential roles in mammalian cells. Ion transport mechanisms in the epididymis regulate sperm volume and fertilization capacity and are critical for the acquisition of sperm motility during maturation (Ren et al., 2025). *TAF7L* has been previously reported to play an important role in spermatogenesis (Moreno-Irusta et al., 2024). Disruptions in this gene have been associated with reduced sperm count and motility in both humans and mice, as well as semen production and scrotal circumference in cattle (de Camargo et al., 2015). Although these traits are phenotypically expressed in males, selection for superior ram fertility may result in detectable signatures on the X chromosome in ewes, as rams transmit their single X chromosome to all female offspring. While sperm quality is critical for fertilization and is a male-specific trait, efficient sperm transport through the female reproductive tract is also essential. For instance, a higher proportion of sperm traversing the cervix and uterus in Belclare ewes led to a greater incidence of fertilization than in a less fertile breed (Fair et al., 2005).

Among the genes detected within the selection signature regions and linked to female reproduction, *MID1IP1* and *IL1RAPL2* have been associated with early embryo development, which may influence traits such as daughter pregnancy rate and productive life (Cole et al., 2011; Shi et al., 2025). Genes belonging to the GABA receptor family, including *GABBR2* (OAR2) and *GABRA3* and *GABRQ* (OARX), were enriched for the GO term GABA receptor activity (GO:0016917). These receptors participate in the neuroendocrine regulation of gonadotropin-releasing hormone (GnRH) in the hypothalamus, influencing luteinizing hormone secretion, corpus luteum function, and consequently progesterone production. Consistent with progesterone's importance for reproductive success, Fair et al. (2007) reported that progesterone concentrations rise earlier in Belclare ewes than in less prolific breeds such as Texel and Suffolk during the periovulatory period, thereby affecting embryo development and subsequent fertility. This aligns with the *FOXO4* gene, detected within a region identified by F_ST_, which may also contribute to reproductive performance, as FOXO transcription factors interact with the progesterone receptor (PGR) to regulate endometrial gene expression and establish a uterine environment that supports early embryo development (Burns et al., 2018).

Selection signatures were detected in autosomal regions harboring genes involved in ovarian function and reproductive regulation. Among them, miR-21, located on OAR11, is a regulatory microRNA implicated in granulosa cell proliferation, follicular growth, and the follicular–luteal transition, playing an important role in follicular development (Yousuf et al., 2025; Zhang et al., 2022). Genes related to hormonal regulation were also detected, including *PAPPA2*, which regulates insulin-like growth factor (IGF) bioavailability and has been associated with ovarian function and litter size in sheep (Wang et al., 2026). *SNORA73* has been suggested to influence ovarian steroid synthesis, further highlighting the potential role of regulatory RNAs in reproductive physiology (Toms et al., 2021). The *EDN2* gene, detected in OAR1, plays a key role in ovulation by triggering follicle rupture in mature follicles (Ko et al., 2022). Together, these findings suggest that selection in the Belclare breed may have targeted genes involved in key ovarian processes, including follicular development, steroidogenesis, and ovulation, contributing to its high prolificacy.

The *ZMYND12* gene, identified within a selection signature region on OAR1, has been associated with sperm flagellar structure and motility, suggesting that genes influencing male fertility may also contribute to reproductive success (Dacheux et al., 2023). Other genes detected in autosomal regions, such as *FOXA2, FUT8*, and *HIVEP3*, are involved in developmental and physiological processes, including uterine gland development, postnatal growth, muscle formation, and bone formation, which may indirectly influence reproductive performance (Kelleher et al., 2017; Purfield et al., 2017; Wang et al., 2006; Zuo et al., 2023). The *RELL1* gene on OAR6 has previously been reported as a candidate gene associated with prolificacy-related traits in livestock species (Wan et al., 2023).

Selection signatures harbored QTL regions associated with offspring number on OAR11 and OARX (Supplementary Table S4), supporting our findings on genes related to fertilization success and greater prolificacy. In addition, QTLs linked to milk production, milk fat, and milk protein were detected on OAR1, 2, 3, 5, 13, 14, and X, highlighting the importance of maternal ability in prolific breeds. As prolificacy alone does not guarantee lamb survival and growth, the capacity to nourish multiple offspring is crucial. The Belclare breed demonstrates advantages not only during the prenatal phase (e.g., ovulation and conception) but also postnatally, with higher lamb survival. This is supported by Shiels et al. (2025) and McHugh et al. (2016), who reported that Belclare ewes experienced fewer dystocia-related lamb deaths and easier lambing than terminal breeds such as Suffolk, Texel, and Vendeen, emphasising the importance of maternal traits in reproductive efficiency.

The present study focuses on detecting signals of positive selection. Therefore, strongly non-favorable alleles for a specific trait under negative selection would not be expected to be detected. Methodological factors such as window size and SNP density can also be a limitation. For instance, the genotyping platform used in this study may limit the detection of low-frequency variants or narrow selection signals. Analyzing sequence data with the same methodologies could capture low-frequency alleles, providing more accurate results and more precise identification of selective signatures, due to the higher genome density and coverage. Using a higher percentile threshold on the X chromosome may increase the risk of false positives. However, cross-validation across XP-EHH and F_ST_, combined with functional annotation, mitigates this risk and allows meaningful identification of selection signatures.

The Belclare breed is genetically distinct from the pooled group of less prolific breeds in our study, as shown in the PCA, which partially mitigates concerns about confounded signals. However, comparing a single prolific breed with a pooled group of less prolific breeds can also be a limitation. While practical and increasing statistical power, this design may conflate true selection signals with signals arising from population structure or breed-specific demographic histories unrelated to prolificacy. Future studies, including additional prolific breeds or refined population controls, could help understand these effects. In addition, the absence of phenotypic data (e.g., litter size and ovulation rate) for the studied animals prevents direct validation of the detected selection signature regions against observed phenotypic variation.

## 5 Conclusions

This study identified selection signatures on autosomes and the X chromosome in Belclare ewes compared with those of less prolific sheep breeds. A common selection signature region was detected on the X chromosome by both methodologies. The genes within these regions were associated with reproductive traits in both ewes and rams. X-linked genes such as *TAF7L, ATP1B4*, and *ZMYND12* were associated with sperm motility, suggesting that selection for ram traits may leave detectable patterns on the ewe’s X chromosome. Genes involved in ovarian processes, including follicular development, steroidogenesis, ovulation, and early pregnancy, such as *MID1IP1, IL1RAPL2*, and *EDN2*, were also identified within the selection signature regions. The Belclare breed demonstrates advantages during both the prenatal phase, such as ovulation and conception, and the postnatal phase, including increased lamb survival. However, these findings are based on in silico associations, and experimental validation, such as gene expression studies and functional assays, is still required before definitive causal roles can be assigned. Overall, the selection signatures detected in Belclare ewes provide a better understanding of their genetic differences in prolificacy compared with those of less prolific breeds, and may be useful for marker-assisted or genomic selection programmes targeting this trait.

## Consent for publication

Not applicable.

## Data statement

The genotype data used in this study cannot be made available by the authors as a third party, Sheep Ireland, manages them. Reasonable requests for genotype data can be made to Sheep Ireland, Ballincollig, Cork, Co. Cork, Ireland: email query@sheep.ie; Fax: +353 (0)238820229; Phone 1850601 901; website www.sheep.ie.

## Funding

This research was supported by the São Paulo State Research Foundation [FAPESP - grant numbers 2022/13986-0], National Council for Scientific and Technological Development [CNPq - grant numbers 130045/2022-5], Coordination for the Improvement of Higher Education Personnel (CAPES, Brasília, Brazil—001 and 88887.802720/2023-00), and 10.13039/501100000023Government of Canada through the ELAP scholarship program.

## Ethical statement

Animal Care and Use Committee approval was not required as the genotypes were from the Irish national sheep breeding program database hosted by Sheep Ireland (http://www.sheep.ie).

## Acknowledgements

The authors acknowledge Teagasc, Animal & Grassland Research for providing the data used in this study and FAPESP, CNPq, and the 10.13039/501100000023Government of Canada for providing the scholarships for JLR to conduct this research.

## CRediT authorship contribution statement

**Julia Lisboa Rodrigues:** Writing – review & editing, Writing – original draft, Visualization, Investigation, Formal analysis. **Flavio Schramm Schenkel:** Writing – review & editing, Supervision, Investigation. **Larissa Graciano Braga:** Writing – review & editing, Investigation. **Ana Carolina Almeida Rollo de Paz:** Writing – review & editing. **Rafael Nakamura Watanabe:** Writing – review & editing. **Noirin McHugh:** Writing – review & editing, Resources. **Donagh Pearse Berry:** Writing – review & editing, Resources, Methodology, Conceptualization. **Marcos Eli Buzanskas:** Writing – review & editing, Supervision, Methodology, Conceptualization. **Danísio Prado Munari:** Writing – review & editing, Supervision, Funding acquisition, Conceptualization.

## Declaration of competing interest

The authors declare that they have no known competing financial interests or personal relationships that could have appeared to influence the work reported in this paper.

## Appendix Supplementary materials

Image, application 1

## References

[bib0001] Abdoli R., Zamani P., Mirhoseini S.Z., Ghavi Hossein-Zadeh N., Nadri S. (2016). A review on prolificacy genes in sheep. Reproduction in Domestic Animals.

[bib0002] Bohan A., Shalloo L., Creighton P., Berry D.P., Boland T.M., O’Brien A.C., McHugh N. (2019). Deriving economic values for national sheep breeding objectives using a bio-economic model. Livestock Science.

[bib0003] Braga L.G., Schenkel F.S., Chud T.C.S., Rodrigues J.L., Saada B., Machado M.A., Panetto J.C.C., da Silva M.V.G.B., Munari D.P. (2025). Selection signatures in Gir and Holstein cattle. Journal of Dairy Science.

[bib0004] Browning B.L., Zhou Y., Browning S.R. (2018). A one-penny imputed genome from next-generation reference panels. The American Journal of Human Genetics.

[bib0005] Burns G.W., Brooks K.E., O’Neil E.V., Hagen D.E., Behura S.K., Spencer T.E. (2018). Progesterone effects on extracellular vesicles in the sheep uterus. Biology of Reproduction.

[bib0006] Cole J.B., Wiggans G.R., Ma L., Sonstegard T.S., Lawlor T.J., Crooker B.A., Da Y. (2011). Genome-wide association analysis of thirty one production, health, reproduction and body conformation traits in contemporary U.S. Holstein cows. BMC Genomics.

[bib0007] Dacheux D., Martinez G., Broster Reix C.E., Beurois J., Lores P., Tounkara M., Dupuy J.W., Robinson D.R., Loeuillet C., Lambert E., Wehbe Z., Escoffier J., Amiri-Yekta A., Daneshipour A., Hosseini S.H., Zouari R., Mustapha S.F.B., Halouani L., Jiang X., Coutton C. (2023). Novel axonemal protein ZMYND12 interacts with TTC29 and DNAH1, and is required for male fertility and flagellum function. ELife.

[bib0008] Davenport K.M., Bickhart D.M., Worley K., Murali S.C., Salavati M., Clark E.L., Cockett N.E., Heaton M.P., Smith T.P.L., Murdoch B.M., Rosen B.D. (2022). An improved ovine reference genome assembly to facilitate in-depth functional annotation of the sheep genome. GigaScience.

[bib0009] de Camargo G.M.F., Porto-Neto L.R., Kelly M.J., Bunch R.J., McWilliam S.M., Tonhati H., Lehnert S.A., Fortes M.R.S., & Moore S.S. (2015). Non-synonymous mutations mapped to chromosome X associated with andrological and growth traits in beef cattle. BMC Genomics.

[bib0010] Fair S., Hanrahan J.P., Donovan A., Duffy P., O’Meara C.M., Lonergan P., Evans A.C.O. (2007). Hormonal relationships during the periovulatory period among ewe breeds known to differ in fertility after cervical artificial insemination with frozen thawed semen. Animal Reproduction Science.

[bib0011] Fair S., Hanrahan J.P., O’Meara C.M., Duffy P., Rizos D., Wade M., Donovan A., Boland M.P., Lonergan P., Evans A.C.O. (2005). Differences between Belclare and Suffolk ewes in fertilization rate, embryo quality and accessory sperm number after cervical or laparoscopic artificial insemination. Theriogenology.

[bib0012] Featherstone N., Hely F.S., McHugh N. (2021). Genetic and economic benefits of foreign sire contributions to a domestic sheep industry; including an Ireland-New Zealand case study. Genetique, Selection, Evolution.

[bib0013] Fonseca P.A.S., Suárez-Vega A., Marras G., Cánovas Á. (2020). GALLO: an R package for genomic annotation and integration of multiple data sources in livestock for positional candidate loci. GigaScience.

[bib0014] Gautier M., Klassmann A., Vitalis R. (2017). Rehh 2.0: a reimplementation of the R package rehh to detect positive selection from haplotype structure. Molecular Ecology Resources.

[bib0015] Gautier M., Vitalis R. (2012). Rehh: An R package to detect footprints of selection in genome-wide SNP data from haplotype structure. Bioinformatics.

[bib0016] Hanrahan J.P., Gregan S.M., Mulsant P., Mullen M., Davis G.H., Powell R., Galloway S.M. (2004). Mutations in the genes for oocyte-derived growth factors GDF9 and BMP15 are associated with both increased ovulation rate and sterility in Cambridge and Belclare sheep (*Ovis aries*). Biology of Reproduction.

[bib0017] Heyer E., Segurel L. (2010). Looking for signatures of sex-specific demography and local adaptation on the X chromosome. Genome Biology.

[bib53] Hill W.G. (1981). Estimation of effective population size from data on linkage disequilibrium. Genet Res.

[bib0018] Hu Z.L., Park C.A., Reecy J.M. (2022). Bringing the animal QTLdb and CorrDB into the future: Meeting new challenges and providing updated services. Nucleic Acids Research.

[bib0019] Keady T.W.J., Hanrahan J.P. (2022). Effects of age at first joining and ewe genotype on the performance of two-tooth ewes and that of their progeny to slaughter. Animals.

[bib0020] Kelleher A.M., Peng W., Pru J.K., Pru C.A., Demayo F.J., Spencer T.E. (2017). Forkhead box a2 (FOXA2) is essential for uterine function and fertility. Proceedings of the National Academy of Sciences of the United States of America.

[bib0021] Kijas J.W., Porto-Neto L., Dominik S., Reverter A., Bunch R., McCulloch R., Hayes B.J., Brauning R., McEwan J., International Sheep Genomics Consortium (2014). Linkage disequilibrium over short physical distances measured in sheep using a high-density SNP chip. Animal Genetics.

[bib0022] Ko C.J., Cho Y.M., Ham E., Cacioppo J.A., Park C.J., Ko C.J., Cho Y.M., Ham E., Cacioppo J.A., Park C.J. (2022). Endothelin 2: a key player in ovulation and fertility. Reproduction.

[bib0023] Kolberg L., Raudvere U., Kuzmin I., Vilo J., Peterson H. (2020). Gprofiler2 – an R package for gene list functional enrichment analysis and namespace conversion toolset g:profiler. F1000Research.

[bib0024] Liu Q., Pan Z., Wang X., Hu W., Di R., Yao Y., Chu M. (2014). Progress on major genes for high fecundity in ewes. Frontiers of Agricultural Science and Engineering.

[bib0025] Mchugh N., Berry D.P., Pabiou T. (2016). Risk factors associated with lambing traits. Animal.

[bib0026] McLaren A., McHugh N., Lambe N.R., Pabiou T., Wall E., Boman I.A. (2020). Factors affecting ewe longevity on sheep farms in three European countries. Small Ruminant Research.

[bib0027] Moreno-Irusta A., Dominguez E.M., Iqbal K., Zhang X., Wang N., Soares M.J. (2024). TAF7L regulates early stages of male germ cell development in the rat. FASEB Journal: Official Publication of the Federation of American Societies for Experimental Biology.

[bib0028] Mullen M.P., Hanrahan J.P. (2014). Direct evidence on the contribution of a missense mutation in GDF9 to variation in ovulation rate of Finnsheep. PLoS One.

[bib0029] Notter D.R. (2008). Genetic aspects of reproduction in sheep. Reproduction in Domestic Animals.

[bib0030] O’Brien A.C., Purfield D.C., Judge M.M., Long C., Fair S., Berry D.P. (2020). Population structure and breed composition prediction in a multibreed sheep population using genome-wide single nucleotide polymorphism genotypes. Animal: An International Journal of Animal Bioscience.

[bib0031] Pokharel K., Peippo J., Honkatukia M., Seppälä A., Rautiainen J., Ghanem N., Hamama T.M., Crowe M.A., Andersson M., Li M.H., Kantanen J. (2018). Integrated ovarian mRNA and miRNA transcriptome profiling characterizes the genetic basis of prolificacy traits in sheep (*Ovis aries*). BMC Genomics.

[bib0032] Purcell S., Neale B., Todd-Brown K., Thomas L., Ferreira M.A.R., Bender D., Sham P.C. (2007). PLINK: A tool set for whole-genome association and population-based linkage analyses. The American Journal of Human Genetics.

[bib0033] Purfield D.C., McParland S., Wall E., Berry D.P. (2017). The distribution of runs of homozygosity and selection signatures in six commercial meat sheep breeds. PLoS One.

[bib0034] Qanbari S., Simianer H. (2014). Mapping signatures of positive selection in the genome of livestock. Livestock Science.

[bib0035] Ren X., Jiang K., Yin J., Ma Z., Chen Z., Yang K., Liu S. (2025). Integrated transcriptomic and metabolomic analysis of goose epididymis reveals molecular markers associated with sperm mobility. Poultry Science.

[bib0036] Rodrigues J.L., Braga L.G., Watanabe R.N., Schenkel F.S., Berry D.P., Buzanskas M.E., Munari D.P. (2025). Genetic diversity and selection signatures in sheep breeds. Journal of Applied Genetics.

[bib0037] Rubin C.J., Megens H.J., Barrio A.M., Maqbool K., Sayyab S., Schwochow D., Andersson L. (2012). Strong signatures of selection in the domestic pig genome. Proceedings of the National Academy of Sciences.

[bib0038] Sabeti P.C., Varilly P., Fry B., Lohmueller J., Hostetter E., Cotsapas C., Xie X., Byrne E.H., McCarroll S.A., Gaudet R., Schaffner S.F., Lander E.S., Frazer K.A., Ballinger D.G., Cox D.R., Hinds D.A., Stuve L.L., Gibbs R.A., Belmont J.W., Stewart J. (2007). Genome-wide detection and characterization of positive selection in human populations. Nature.

[bib0039] Saravanan K.A., Panigrahi M., Kumar H., Bhushan B., Dutt T., Mishra B.P. (2020). Selection signatures in livestock genome: A review of concepts, approaches and applications. Livestock Science.

[bib0040] Shi M., Li G., Araujo H.M., Lee A.S., Zhang J., Lee Y.L., Consortium IISAGE, Cheong S.H., Duan J.J.E. (2025). Sex-biased transcriptome in in vitro produced bovine early embryos. Cell & Bioscience.

[bib0041] Shiels D., Mee J.F., Hanrahan J.P., Dwyer C.M., Fagan S., Keady T.W.J. (2025). Timing, risk factors, and causes of foetal and preweaning lamb mortality in lowland production systems involving a range of ewe genotypes. Animal.

[bib0042] Toms D., Pan B., Bai Y., Li J. (2021). Small RNA sequencing reveals distinct nuclear microRNAs in pig granulosa cells during ovarian follicle growth. Journal of Ovarian Research.

[bib0043] Wan X., Jing J.N., Wang D.F., Lv F.H. (2023). Whole-genome selective scans detect genes associated with important phenotypic traits in goat (*Capra hircus*). Frontiers in Genetics.

[bib0044] Wang X., Gu J., Miyoshi E., Honke K., Taniguchi N. (2006). Phenotype changes of Fut8 knockout mouse: Core fucosylation is crucial for the function of growth factor receptor(s). Methods in Enzymology.

[bib0045] Wang Y., Xu W., Liang B., Li S., Hu W., Chu M. (2026). Comprehensive analysis of PAPPA2 expression and genetic variants in sheep and their association with litter size. Veterinary Medicine and Science.

[bib0046] Weir B.S., Cockerham C.C. (1984). Estimating F-statistics for the analysis of population structure. Evolution.

[bib0047] Wright S. (1949). The genetical structure of populations. Annals of Eugenics.

[bib0048] Yousuf S., Malik W.A., Feng H., Liu T., Xie L., Miao X. (2025). Integrated analysis of microRNA and mRNA interactions regulating fecundity in the ovaries of two distinct sheep breeds. BMC Genomics.

[bib0049] Zhang H., Liu A., Wang Y., Luo H., Yan X., Guo X., Li X., Liu L., Su G. (2021). Genetic parameters and genome-wide association studies of eight longevity traits representing either full or partial lifespan in Chinese Holsteins. Frontiers in Genetics.

[bib0050] Zhang T., Huo S., Wei S., Cui S. (2022). miR-21, miR-125b, and let-7b contribute to the involution of atretic follicles and corpus lutea in Tibetan sheep ovaries. Animal Science Journal.

[bib0051] Zhu C., Fan H., Yuan Z., Hu S., Zhang L., Wei C., Zhang Q., Zhao F., Du L. (2015). Detection of selection signatures on the X chromosome in three sheep breeds. International Journal of Molecular Sciences.

[bib0052] Zuo P., Zhang C., Gao Y., Zhao L., Guo J., Yang Y., Yu Q., Li Y., Wang Z., Yang H. (2023). Genome-wide unraveling SNP pairwise epistatic effects associated with sheep body weight. Animal Biotechnology.

